# Communicating Program Outcomes to Encourage Policymaker Support for Evidence-Based State Tobacco Control

**DOI:** 10.3390/ijerph111212562

**Published:** 2014-12-04

**Authors:** Allison M. Schmidt, Leah M. Ranney, Adam O. Goldstein

**Affiliations:** Tobacco Prevention and Evaluation Program, Department of Family Medicine, University of North Carolina at Chapel Hill, 590 Manning Drive, Chapel Hill, NC 27599, USA; E-Mails: Leah_Ranney@unc.edu (L.M.R.); adam_goldstein@med.unc.edu (A.O.G.)

**Keywords:** tobacco smoking, tobacco control policies, communication, public health

## Abstract

Tobacco use, the leading cause of preventable death in the U.S., can be reduced through state-level tobacco prevention and cessation programs. In the absence of research about how to communicate the need for these programs to policymakers, this qualitative study aimed to understand the motivations and priorities of policymakers in North Carolina, a state that enacted a strong tobacco control program from 2003–2011, but drastically reduced funding in recent years. Six former legislators (three Democrats, three Republicans) and three lobbyists for health organizations were interviewed about their attitudes towards tobacco use, support of state-funded programs, and reactions to two policy briefs. Five themes emerged: (1) high awareness of tobacco-related health concerns but limited awareness of program impacts and funding, (2) the primacy of economic concerns in making policy decisions, (3) ideological differences in views of the state’s role in tobacco control, (4) the impact of lobbyist and constituent in-person appeals, and (5) the utility of concise, contextualized data. These findings suggest that building relationships with policymakers to communicate ongoing program outcomes, emphasizing economic data, and developing a constituent advocacy group would be valuable to encourage continued support of state tobacco control programs.

## 1. Introduction

Tobacco use remains the single most preventable cause of disease, disability, and death in the U.S. [1]. Prevention and cessation are critical, as 87% of smokers start smoking before the age of 18 [1], and still about one-fifth (18.1%) of the U.S. population smokes [2]. Prevention and cessation are achievable, particularly through state-level programs, legislation, and policies, including youth tobacco prevention programs, media campaigns, and telephone quitlines [3]. In many states, legislators have reduced tobacco control program funding, despite evidence that programs are effective at reducing tobacco use nationally [4,5,6,7] and within North Carolina [8], and that they generate financial return on investment to states [9]. The current study seeks to understand state policymakers’ views and priorities to facilitate better communication between public health program evaluators and policymakers responsible for allocating program funding. North Carolina is seen as a particularly relevant setting for this line of inquiry, as it is a state that had successful tobacco prevention and cessation programs [10], but has in recent years dramatically cut funding [3]. 

At the state level, funding for tobacco control programs is available through several sources, including the Master Settlement Agreement (MSA), revenue generated from taxes on tobacco products, and other general revenue streams [3]. In 1998, state Attorneys General across the country sued the tobacco industry for costs associated with tobacco-related diseases, resulting in the MSA, a 25-year payout of hundreds of billions of dollars to states to offset Medicaid costs and provide education to reduce youth smoking [11]. However, funds were not earmarked for these purposes; individual states were given authority to determine their allocation [11]. 

In many states, funds that have been historically used for tobacco control programs have been diverted for other uses [3]. In North Carolina, such funds were used initially to establish successful statewide programs, including Tobacco Reality Unfiltered (TRU), a teen tobacco prevention program with a media campaign and youth coalition activities, as well as the state telephone quitline to promote smoking cessation [8,10]. Over time, the state diverted this money from tobacco control to close budget deficits [10].

As funding for state programs to prevent and reduce tobacco use is in jeopardy across the country [1,3], it is critical for public health advocates to effectively communicate program results to policymakers. Existing studies of communication to policymakers emphasize the importance of personal relationships and direct communication to convey research findings to policymakers in a useful way [12,13]. Relationships with constituents are especially important; educating and encouraging advocacy among constituents on public health issues can influence policymakers’ priorities [14]. In dissemination efforts, summaries of evidence that are concise, are solution-oriented (not merely critical of a particular policy), and quantify impacts of different policy options are particularly valuable [15], as are proactive and timely reports of tobacco control program successes [16]. Overall, understanding policymakers’ needs and perspectives is critical to generating and delivering an effective policy-related message [15]. While some studies [17,18,19] have examined policymakers’ attitudes towards tobacco control, few have explored ways to inform the decision process for supporting evidence-based tobacco prevention and cessation programs, especially in the current economic context.

The current research was conducted to better understand the views, motivations, and priorities of those integral to tobacco control funding decisions in North Carolina, and how to facilitate communication of tobacco control program results. Findings from this research show how public health practitioners may communicate program outcomes, while being responsive to the needs and values of the policymakers themselves, to promote evidence-based tobacco control policies. 

## 2. Methods and Materials

All subjects gave their informed consent before participating in the study. This study was conducted in accordance with the Declaration of Helsinki, and the protocol was approved by the Institutional Review Board at the University of North Carolina at Chapel Hill (Study #12-0582).

### 2.1. Participants

Participants included former state senators and representatives, and current lobbyists from health-related organizations who communicate with legislators on issues of public health. Former legislators were recruited from a list of 33 Democratic and 11 Republican legislators who served in the 2009–2010 general session but who were not currently members of the legislature. Lobbyists for health-related organizations were recruited from a list of 13 individuals recommended by the North Carolina Alliance for Health, a statewide policy advocacy coalition. Individuals on each of the three lists were ranked in order of effectiveness, as determined by the North Carolina Center for Public Policy Research [20,21], to ensure that the opinions expressed in interviews were representative of policymakers deemed to be influential. Potential participants were called by phone for participation in order of effectiveness ranking. 

The project goal was to recruit the three highest ranking Democrats, Republicans, and lobbyists that would agree to share their thoughts on statewide tobacco prevention and control programs. Successful contact was made with eight legislators and three lobbyists, of which six legislators and all three lobbyists subsequently agreed to participate in an in-person or telephone interview. One Democrat and one Republican legislator declined to be interviewed. The final sample included three Democrats (all former senators), three Republicans (two former senators, one former representative), and three lobbyists. As a token of appreciation for their time, participants were offered a small gift from United Way.

### 2.2. Interviews

State legislators and lobbyists were interviewed about their attitudes and beliefs about tobacco prevention and cessation programs, and their reactions to two policy briefs that framed program outcomes in terms of either lives or dollars saved. In-person interviews were conducted with two legislators and two lobbyists in office settings and coffee shops. Five interviews were conducted by phone, with policy briefs emailed to participants prior to the interview. The order in which participants viewed the two briefs was counterbalanced to limit the potential for order effects. Interviews lasted for approximately 30–45 min and were conducted by one member of the research team. All interviews were audio-recorded with the participants’ permission and transcribed.

### 2.3. Interview Guide Development

Interview guide development was informed by health behavior and communication theories. The Integrated Behavioral Model (IBM) posits that behavior is influenced by knowledge and skills to perform a behavior, environmental factors, the salience of the behavior, habit, and behavioral intention, which itself is determined by attitudes, perceived social norms, and feelings of personal agency or self-efficacy [22]. In our interview guide, we addressed legislators’ knowledge and attitudes about supporting state tobacco control programs, perceived norms of support among members of their political party, and environmental factors that may affect their willingness to support tobacco prevention and cessation initiatives. We also asked about factors identified in past research as likely influences on policymaker behavior, including political ideology [17,19], interactions with lobbyists and constituents [23], and personal exposure to the negative effects of tobacco use [17]. Probing questions were developed to further explore participant responses. The interview guides were reviewed by several content experts and revised based on their feedback. The guides were pilot tested in preliminary interviews, and minor wording clarifications were made.

### 2.4. Policy Brief Development 

Two policy briefs were developed using purposefully different framing and narrative elements, informed by communication theory and evidence about policymakers’ attitudes towards tobacco control. As the way in which a message is characterized or presented can have a significant effect on how it is perceived [22], one policy brief was framed in terms of saving lives, and the other in terms of saving dollars. Additionally, the “Saving Lives” brief incorporated real narratives of young people touched by tobacco use or participation in tobacco prevention initiatives (as well as some key facts about program participation and changes in smoking), as narratives may be more compelling and persuasive than facts alone [24,25,26,27]. The “Saving Dollars” brief presented data on costs saved by the programs and potential financial costs of discontinuing funding with no narrative elements. Policy brief content was informed by prior literature on communicating to policymakers in that it delivered concise, quantifiable effects of existing tobacco prevention and cessation programs [15]. 

### 2.5. Qualitative Data Analysis

Interviews were transcribed with identifying information removed and coded by two researchers. A codebook was jointly developed to include deductive codes, based on constructs from the interview guide, and inductive codes, based on the content of participants’ responses. The two researchers compared their coding of randomly selected sections of the interviews and found their coding to be very consistent. Minimal differences were resolved by clarifying in the codebook specific subjects for which a particular thematic code should or should not be used. Coded sections of each interview were compiled and major themes were identified. Participants reported agreement with interview summaries that described their responses in each of the themes.

## 3. Results

Five themes emerged from the interviews: (1) high awareness of health concerns but limited awareness of program impact and funding sources, (2) the primacy of economic concerns in the state legislature and utility of economic data in policy briefs, (3) ideological differences in views of the state’s role in tobacco prevention, (4) the impact of lobbyist and constituent appeals, and (5) the utility of concise, contextualized messages. These themes were identified as areas that shape policymakers’ support for tobacco prevention and cessation programs and that reflect shared views about effective policy communication.

### 3.1. High Awareness of Health Concerns but Limited Awareness of Program Impact and Funding Sources

Legislators seemed aware of the extent of the problems presented by tobacco use. They articulated how the health implications of tobacco use and resulting medical costs placed a heavy burden on both individual citizens and the state.

*“We can’t fund every health need we have, but tobacco consumption is so—again, we know the ills and the costs are so pervasive. It’s kind of different than anything else I can think of that’s manageable, that’s preventable.” (Republican)*

Despite knowledge of health issues caused by tobacco use, legislators indicated they knew relatively little about the effectiveness of the state’s current tobacco prevention and cessation initiatives. They felt that knowing the impact of current programs and the return on investment of prior program spending was necessary in order to prioritize such programs for future funding.

*“I want to see some results of what we have done so that I can evaluate whether we need to do more of it or less of it or change things around. If the data is showing that we’ve got 80% fewer people smoking than we did before that’s a good thing. […] That’s the kind of thing that I think legislators are going to be looking for with anything that comes out.” (Democrat)*

Several participants seemed to have only limited knowledge regarding the details of the MSA that had funded the current programs. While many supported honoring the original intentions of the agreement, few knew the specific terms of the agreement, the extent of funds available, or where the majority of funds were currently being spent.

*“I understood that [the Health and Wellness Trust Fund was] where the tobacco settlement money was supposed to go, or part of it. And I also have a recollection […] that some of that money that was supposed to have gone there has gone to other places, which is always concerning. It’s not atypical of politics to see that happen. […] I don’t know what size it is. I don’t know how much funds were diverted to other places from that.” (Republican)*

### 3.2. Primacy of Economic Concerns

Based on their knowledge of the health and financial costs of tobacco use, all participants generally supported the idea that tobacco prevention and cessation programs improve health for North Carolina’s residents. However, their primary concern was to spend the state’s limited dollars in a responsible way. Participants viewed spending for tobacco control as being pitted against spending for other priorities, such as education. Legislators spoke at length about their responsibility to constituents to prioritize and balance these competing needs in a manner that yielded the most good for the state.

*“[T]o get a budget appropriation in this time to sustain the state programs would be difficult. I mean, I wouldn’t vote against it, but I’d have to prioritize […] and I’d have to say, “Okay, am I gonna vote for a tuition increase at Chapel Hill or am I gonna vote to put some money into the program to try to keep people from smoking?” And I would say that holding back tuition increases at Chapel Hill is more important.” (Democrat)*

The importance of economic concerns was also apparent when participants chose which policy brief they preferred. Six out of seven participants who preferred one brief over the other preferred the Saving Dollars brief. When asked why they chose the Saving Dollars brief over the Saving Lives brief, responses included:

*“I guess it’s because it’s dealing with the money and everybody is focused these days on the economy and I guess that’s kind of on my brain right now.” (Democrat)*

*“I would say probably the first one [Saving Dollars], the impact on the Medicaid costs and healthcare costs because we’re all concerned about that.” (Democrat)*

*“I probably like this one [Saving Dollars] better because it’s directly on point. This one [Saving Lives] says that fewer kids are smoking and we know that smoking is bad. But it doesn’t tell me what caused it or what return on our investment we get for our tax dollars. This one [Saving Dollars] very specifically does.” (Republican)*

Of particular interest to the legislators were facts presented related to tobacco prevention and cessation programs and Medicaid. Policymakers showed a high level of interest in controlling Medicaid costs, indicating that even the mention of saving Medicaid dollars in a policy brief would get the attention of state legislators.

*“I would just focus on the Medicaid costs and healthcare costs and that kind of stuff. I think that makes a bigger statement than anything.” (Democrat)*

*“It’s like, Oh, wait a minute. They’re talking about Medicaid money. What’s this about?” (Republican)*

### 3.3. Ideological Differences in Views of the State’s Role in Tobacco Prevention 

While interviewees generally agreed that the state needed to address tobacco-related issues, they disagreed about what this role entailed. Ideological perspectives about individual responsibility for health were often at the center of this debate. Among our interviewees, both Democrats and Republicans emphasized individual responsibility for tobacco use. Several participants suggested alternative strategies to reduce tobacco use, including supporting incentives for donations to nonprofit organizations rather than directly funding state programs. These same individuals also acknowledged that some level of state support would probably be necessary, however, to complement such efforts.

*“First of all, we want from a policy standpoint, to see interest groups that may take on projects…like helping people stop smoking. We want to see those groups thrive and be active and receive funding from donors and would give tax incentives to the donors to fund them...So, number one, I prefer that type of approach. Secondly though, our societal costs from the use of tobacco are so horrendous that probably something more has to be done.” (Republican)*

### 3.4. Lobbyist and Constituent Advocacy 

Several legislators mentioned the value of information shared by lobbyists, acknowledging that it is not possible to know about every subject area on which they might be asked to make decisions. However, they also noted that they did not immediately take facts presented by lobbyist at face value but instead verified the source of the data presented or actively sought additional information.

*“Lobbyists are great resources in Raleigh and they’re very underappreciated by the public. The public somehow looks at them negatively. They really should look at them positively. Otherwise they're just legislators up there making big decisions with absolutely no time or resources to research the sides of the issue.” (Republican)*

While lobbyists played important roles, former legislators stated that they valued input from their constituency even more. Messages were most effective when delivered in person by individuals directly impacted by tobacco, especially survivors of tobacco use from a legislator’s home district. Lobbyists echoed the need for personal contact from constituents in combination with other advocacy activities.

*“[I]f the group can find some individuals, in their districts or in the state, who had a family member who, for example, had emphysema or lung cancer, cancer of the esophagus or whatever, and just let that legislator know, ‘This is what tobacco use did to my loved one,’ something like that can really be effective and, ‘Please help us support this program so people do not have to suffer from this preventable disease.’” (Democrat)*

*“You always want to hear from your constituents […] People probably don’t realize how much their communications work, especially when they’re not coordinated and not all just replications of the same wording and that type thing. […] They get your attention. So I think they’re very effective.” (Republican)*

*“If I were to receive a policy brief, if I were a legislator…I think it would be helpful if it was delivered to me by one of my constituents, with a follow up by paid lobbyists or citizen lobbyists.” (Lobbyist)*

*“I think things that stand out include presentations, people who come to Raleigh, and are able to get before us and tell us so we see people with their own stories, whether it be someone who had part of their esophagus removed or trachea from smoking or whatever. I think those things are very effective. You remember those people that come to Raleigh that are there.” (Republican)*

### 3.5. The Utility of Concise, Contextualized Messages

When policy briefs were presented, interviewees emphasized the importance of succinct information. Given the volume of material coming through their office and their limited time, interviewees valued the policy briefs.

*“You’ve got the road issues, and you’ve got education issues, you’ve got justice and public safety issues, and parole issues, and then some constituent that needs help with her department and in the meantime, you’re supposed to read this stuff, retain it and go to the next thing.” (Republican)*

*“If you give them multiple pages, they just don’t have time to read it. They want you to synthesize your argument. So I put stuff in bullets […].” (Lobbyist)*

While having a nice appearance for the policy brief was important, one legislator and one lobbyist described a fine line between attractiveness and appearing overly expensive to produce. While not discussed broadly among interviewees, materials that appeared expensive to produce may raise questions to why funding was diverted from programs to produce policy briefs.

*“Not real fancy, I like the simple. [Inaudible] doesn’t look costly, but some groups spend money on messaging instead of services […] so I like the fact it doesn’t look expensive.” (Republican)*

*“And you don’t want to look too slick, though […] that fine line between making it look really good and making it too much, too expensive.” (Lobbyist)*

Interviewees indicated that merely presenting facts and statistics was insufficient. Several participants mentioned wanting numerical data to be put into context. They felt this would help them to digest the statistics and appreciate and understand their relevance. Participants suggested that different types of information would help to do this, including showing other ways the money saved could be spent, providing a greater sense of the scope of the problem, such as how many smokers there are in the state, or showing the change in tobacco use over time.

*“[I]f you just throw out figures it gets mixed up in other figures to the point that it just doesn’t mean anything. You need another way of saying that this is saving $928 million dollars in medical costs. Make it so that I know what $928 million dollars means […] [I]f you turn that around and you say for $928 million dollars we could send so many kids to college; or if you say $55 million dollars in medical costs we could provide lunch for 10,000 students throughout the state, or just something that I can relate to it’ll mean something to me. But if you just give me broad figures like that it just doesn’t register.” (Democrat)*

## 4. Discussion

Across the country, states have drastically reduced funding for tobacco control programs from MSA monies and other sources, despite having the resources and the evidence of effectiveness needed to continue them [3]. In 2014, states spent just 1.9% of the revenue collected from the tobacco industry on tobacco control, continuing a downward trend in spending over the past several years [3]. In this climate, it is imperative for researchers and public health practitioners to communicate to policymakers in a way that drives the development of evidence-based state tobacco control, as the costs of not funding such programs are dire in terms of lives and money [1]. Findings from this research suggest some key strategies for public health professionals to communicate program evaluation results to policymakers.

First, stronger education of policymakers about existing tobacco control programs results is needed. Interview participants showed high awareness of the health risks of tobacco use but limited understanding of the MSA or outcomes of existing tobacco control programs, consistent with findings in past research [12,15,18]. These findings suggest that public health professionals should foster ongoing communication with policymakers about continued successes of state programs and not initiate contact only when funding is jeopardized. Prior research too supports the importance of proactive, timely communication of evaluation data [28], as well as relationship-building between researchers and policymakers to promote policymaker support for tobacco control [14,16]. By strengthening relationships with policymakers, researchers can both better communicate their own messages and better meet the informational needs of individual policymakers as well. 

Second, emphasizing economic data may be of particular importance in communications to policymakers. Policymakers expressed the difficulties of spending limited resources in the most fiscally responsible ways possible across a range of priorities. Participants emphasized that tobacco control messages should highlight economic data, particularly Medicaid costs, should be brief and concise, and should put statistics into understandable contexts for maximum message impact. A recent study of state policymakers in Nevada similarly showed policymakers had substantial knowledge of the hazards of smoking, yet did not support strengthening the Nevada Clean Indoor Air Act, in large part due to economic concerns of hurting small gaming businesses [29], however unlikely these concerns may have been [30]. Emphasizing the return on investment of tobacco control programs, and, to the extent possible, that negative business impacts are unlikely to occur, seems especially critical in an economic climate of lower budgets overall to fund multiple important programs.

Third, educating and involving constituents in advocacy for strong and continued tobacco control are important to promote policymaker support. Relationships with constituents were noted as a key influence on policymakers’ priorities, as has been found in other research as well [14,23,29]. For example, among Nevada state legislators, a majority stated that they would vote to strengthen the state’s clean indoor air laws if their constituents supported it, despite other concerns [29]. With respect to message delivery, participants also said that in-person appeals, especially from constituents who had experienced the harms of tobacco use, would be particularly effective at garnering their support for tobacco prevention and cessation programs. These results suggest that a stronger tobacco victim and survivor advocacy network, with representation from multiple legislative districts and coordinated with in-person delivery of policy briefs, may facilitate support for tobacco prevention and cessation programs [17,31].

Fourth, it may be helpful to frame the benefits of tobacco control programs in a way that is grounded in the core values espoused by the policymakers themselves, many of whom emphasized individual responsibility for tobacco use [17,18,19]. Such messages could emphasize values tied to individual freedom, such as the importance of individual freedom from the tobacco industry and addiction, and free enterprise as being compromised by tobacco-related illnesses [18]. Additionally, education of policymakers, and perhaps the public, about the environmental and social drivers of smoking, and how existing programs change these factors may be beneficial as well. Support for this recommendation was found in a study of Kansas legislators with respect to nutrition and physical activity-related policies, two other issues frequently considered to be an individual’s responsibility [32]. Furthermore, prior research shows the most widespread support across policymakers for tobacco prevention among youth, suggesting program benefits to young people may be an especially good focus for a message [18]. Important messages, given that smoking is perceived as an issue of individual responsibility, are that tobacco control programs can help support individual freedoms, particularly for youth, by affecting the environmental and social drivers of smoking.

There were several limitations to this study. First, while the sample size was small, it was selected as adequate for this qualitative exploratory study. Still, conclusions cannot be generalized as being applicable to all former legislators and lobbyists in the state, nor to those who are currently in the state legislature at any given time. Another limitation is that state funding in the interview guide was conceptualized as MSA monies, which is not the only source of funds available to states for tobacco control. Last, as North Carolina is a top tobacco producing state, its legislators may be somewhat more hesitant to support tobacco control than the average state. However, the key lessons learned from this study about useful strategies for policymaker communication seem broadly applicable and consistent with past research done in other states as well.

It seems that some states are already using strategies consistent with this report [16]. For example, New York State, in its sending of a concise, accessible tobacco control program evaluation report to every state legislator [33] and garnering wide media coverage, has sustained successful tobacco control programs [16], as had North Carolina [34,35] until a large part of its funding mechanism, the Health and Wellness Trust Fund, was discontinued [36]. As a next step, practitioners and researchers should more widely test our recommendations and those found in the literature to identify best practices and successfully securing funding for critical state tobacco control programs.

## 5. Conclusions

The current study is consistent with prior work and extends insights into how to best communicate with policymakers to support the development of evidence-based tobacco control, especially in the current economic and political environment. Such work is critical, as the costs of not funding tobacco control programs far outstrip the costs of funding them [6,7,37]. Four key recommendations for action by public health program evaluators emerged from our work: (1) build ongoing relationships with policymakers to communicate tobacco control program results, (2) emphasize economic data in such communications, (3) educate and involve constituents in advocacy for continued tobacco control, and (4) frame the benefits of tobacco control programs as being grounded in the core values held by policymakers. In this way, public health practitioners can convey important program outcomes to facilitate the development and continuation of evidence-based public health policy, while being responsive to the informational needs and priorities of policymakers themselves.
